# “What Would You Do?”: How Cat Owners Make End-of-Life Decisions and Implications for Veterinary-Client Interactions

**DOI:** 10.3390/ani11041114

**Published:** 2021-04-13

**Authors:** Katherine Littlewood, Ngaio Beausoleil, Kevin Stafford, Christine Stephens

**Affiliations:** 1 Animal Welfare Science and Bioethics Centre, School of Veterinary Science, Massey University, Palmerston North 4442, New Zealand; N.J.Beausoleil@massey.ac.nz; 2 Animal Science, School of Agriculture and Environment, Massey University, Palmerston North 4442, New Zealand; k.j.stafford@massey.ac.nz; 3 School of Psychology, Massey University, Palmerston North 4442, New Zealand; C.V.Stephens@massey.ac.nz

**Keywords:** cats, veterinarians, quality of life, animal welfare, euthanasia, end of life, decision making, validation

## Abstract

**Simple Summary:**

Cats are the most popular companion animals in New Zealand. Many owners form close relationships with their cats and these relationships, along with other factors in cat owners’ lives, make end-of-life decisions complicated. Veterinarians can help make owners’ end-of-life decisions easier if they understand the personal factors impacting such decision making. We set out to explore how owners of older and chronically ill cats make end-of-life decisions in New Zealand and the role that their veterinarian plays in the process. We interviewed cat owners who had recently had their cat euthanized. Cat owners mentioned nine areas of concern. Four were animal-centered concerns: cat behavior change, pain, signs of ageing, and the benefits of having an outside perspective. Five were human-centered concerns: veterinarians understanding owners’ relationships with their cat, normalizing death, the need for a good vet to manage end of life, veterinary validation that owners were doing the right thing, and a desire to predict the time course and outcome for their cat. End-of-life decision making is complex, and the veterinarian’s role is often poorly defined. Our owners appreciated the expertise and validation that their veterinarian provided.

**Abstract:**

Cats are the most common companion animals in New Zealand. Advances in veterinary care means that cats are living longer and there are many older cats. End-of-life decisions about cats are complicated by owner–cat relationships and other psychosocial factors. Our study explored the ways in which end-of-life decisions were being made by owners of older and chronically ill cats in New Zealand and the role of their veterinarian in the process. Qualitative data were gathered via retrospective semi-structured interviews with 14 cat owners using open-ended questions. Transcripts of these interviews were explored for themes using template analysis and nine themes were identified. Four were animal-centered themes: cat behavior change, pain was a bad sign, signs of ageing are not good, and the benefits of having other people see what owners often could not. Five were human-centered themes: veterinarians understanding owners’ relationships with their cat, normalizing death, the need for a good veterinarian to manage end of life, veterinary validation that owners were doing the right thing, and a strong desire to predict the time course and outcome for their cat. End-of-life decision making is complex, and the veterinarian’s role is often poorly defined. Our owners appreciated the expertise and validation that their veterinarian provided but continuity of care was important. Future research aimed at exploring the veterinarian’s perspective during end-of-life decision making for cats would be a valuable addition to the topic.

## 1. Introduction

Cats have become increasingly popular companions [1], and are the most common companion animal (CA) in New Zealand [2]. The increase in cat numbers may be due to some changes in human lifestyle which restrict the keeping of dogs (e.g., renting properties). In addition, as veterinary care available for cats has improved, many cats are living longer [3,4]. This has led to an increase in the number of old cats [2,5,6]. Coupled with a life expectancy that exceeds that of most dog breeds and a propensity to develop chronic diseases in older age [7,8], older cat welfare is a pressing concern. 

Decisions about how the end of animals’ lives should be managed are an important influence on animal welfare. The process of decision making about cat euthanasia is a component of end-of-life (EoL) management, alongside managing the euthanasia process itself and grief, and often involves the veterinarian in consultation with the animal’s owner. Knowledge of how owners make EoL decisions for their cats, and the role of their veterinarian in the process, is important for the animal, veterinarian, and client. It is essential for good animal welfare that the cat is killed using a properly applied, humane method within an appropriate timeframe, to minimize suffering [9].

For the veterinarian and their profession, there are personal, economic, and reputational implications that may influence whether a client returns to the practice following the euthanasia [10,11,12,13,14,15]. Veterinary medicine, in contrast to the majority of human medicine practice, allows for, and often encourages, euthanasia as an option for EoL management [9]. The definition of euthanasia typically encompasses both the application of a technique which minimizes suffering and killing being performed for the animal’s benefit [9,16]. The New Zealand Code of Professional Conduct for Veterinarians expressly mentions the ‘duty’ of veterinarians ‘to protect animal welfare and alleviate animal suffering’ [12]. In addition, carrying out a job ‘well’ also engenders a sense of pride that contributes to workplace satisfaction [17,18], which in turn is important for personal well-being [19,20,21]. Accordingly, competency in EoL management is expected of veterinarians, and directly benefits veterinarians and their profession [12]. 

In Western jurisdictions, animals are considered the property of their owners—for example, in The New Zealand Animal Welfare Act 1999 [22]. This has implications for EoL management because the owner is legally entitled to decide whether and when to end the life of their animal. The EoL process, and how it is managed by the attending veterinarian, can also affect owners [23,24,25]. Cat owners may experience profound grief over the loss of their cat, and anticipatory grief can result in delayed euthanasia [14,26,27]. Owners may also experience guilt before and after euthanizing their cat [28,29]. Veterinarians need to understand these and other psychosocial factors to communicate effectively with clients during the EoL process [29,30]. Negative experiences with veterinarians during EoL management may result in the client telling family and friends about their grievances or, in extreme circumstances, taking the matter further and making a formal complaint to the relevant veterinary regulatory authority [31]. Conversely, veterinarians who are able to understand their clients’ perspectives and how their decisions are made will be best placed to help them come to a decision that benefits all three parties: veterinarian, client, and cat.

Previous studies have reported a number of factors that are key to owners’ EoL decisions but the way the data were collected and analyzed may not represent owners’ holistic experiences of the process and their interactions with veterinarians. For example, factors identified from previous research include the animal’s quality of life (QoL), the owner’s finances, time and other resources, and their attachment to their cat [32,33,34,35]. Many of the factors in other previous studies were identified via ‘expert’ consensus [36], or used in surveys [29,37], and therefore the information collected may not reflect owner perceptions but expert consensus, or has been limited to quantitative data via surveys or structured interviews. For example, Slater at al. [38] interviewed owners of cats with cancer before and after a euthanasia decision. However, their interviews were structured and the resulting quantitative data were analyzed via logistic regression [38]. The problem with collecting quantitative data to explore owner EoL decision making is that it uses a deductive approach. Participants are given pre-conceived options to choose from by the researchers, rather than being given the opportunity to express their own ideas. Methods to collect qualitative data may address some of these limitations.

The few studies that used open-ended inductive (exploratory) methods to collect qualitative data found a number of factors important to owners facing the death of their CA. For example, in a study analyzing narratives written by Finnish CA owners about their experiences with animal death, Schuurman [39] found that euthanasia location, grief experiences, and how the animal perceived the euthanasia were important to owners. Schuurman’s [39] study is limited as it relies on unidirectional information transfer and lacks the in-depth and back-and-forth discussion inherent in interview-based research. Christiansen et al. [40,41] used interviews to collect prospective qualitative data from owners considering treatment or euthanasia of their chronically ill or aged dogs. They found that decision making was more challenging for dog owners if their dog appeared normal or deteriorated slowly, they were unable to clearly assess their dog’s QoL, or if owners had conflicting concerns (e.g., prioritising owner versus dog, or quantity versus quality of life). The veterinarian’s role in decision making also varied by client and was dependent on owner preferences for veterinary involvement [40]. Christiansen et al.’s [40] focus was on the veterinarian’s role in the process, as perceived by their client, and they did not ask questions about how owners made the decision. They also never explicitly asked owners how they assessed the QoL of their dog [40]. Stoewen et al. [42,43,44] also interviewed dog owners, rather than cats. Their participants were clients seeking cancer treatment and wanted to be given honest and detailed information about their dog. This information allowed their participants to trust their veterinarian enough to engage in treatment and be prepared for the future, while also engendering a sense of control to the situation.

Retrospective interviews with CA owners have tended to focus on the grief experience, rather than the EoL decision-making process [45,46]. Additionally, the focus of previous work has been on CAs in general [45,46], or dogs in particular [40,41,42,43,44]. Very few studies have focused explicitly on EoL decision making for felines [47], and none have done so using a retrospective exploratory methodology in the form of in-person client interviews. To begin to bridge the gaps in existing knowledge, an inductive methodology using interviews to gather qualitative data was used in this research. The process of EoL decision making is fraught with challenges and many potentially complicating factors exist. Therefore, it follows that qualitative data best reflect the essence of the decision making process for cat owners [48,49].

The aim of this study was to explore the ways in which EoL decisions were made by owners of older and chronically ill cats in New Zealand and the role of their veterinarian in the process. The potential outcomes of this study are a more formal understanding of cat owner EoL decision making and owner–veterinarian interactions. 

## 2. Materials and Methods 

### 2.1. Rationale for Research Approach

This research is grounded in a constructivist epistemological position, that is, it seeks to examine a particular social situation and achieve a holistic understanding of others’ experiences [49,50]. This is in contrast to the quantitative, hypothesis-driven approach used to explore this topic by previous researchers which may have limited our understanding of this complex situation by excluding an exploratory methodology. This social science approach is more likely to produce the rich data needed to address the aims of this research. 

We recognize that knowledge reflects our perspectives as researchers and cannot be separated from this to create ‘objective’ data. Thus, we acknowledge that the information obtained here has been formed as a result of the research process and that we have had an active role in creating it [49,51,52]. 

As veterinarians and cat owners, the first and third authors have personal experience of some of the beliefs and practices explored in this project. We have taken an inductivist (theory building) approach to collecting and analyzing the data; it was our intention to draw conclusions from the data and explore alternative reasoning, rather than test pre-conceived theory [50,52,53]. 

### 2.2. Study Design

This research project was reviewed and approved by the Massey University Human Ethics Committee Southern B, Application 16/43. Over a 7-month period (May to December 2018) interviews were conducted with New Zealand cat owners who had recently had their cats euthanized. Participants were recruited via social media advertising and provided written consent to participate in this study.

At the time of recruitment, potential interviewees were asked to complete an online recruitment questionnaire via Qualtrics survey software. The recruitment criteria for inclusion in this study were that the individuals lived in New Zealand, were over 18 years of age, had made the decision to euthanize their own cat within the last 6 months, agreed to us interviewing the veterinarian involved in the euthanasia of their cat, and provided contact details for themselves and their veterinarian. Additional information collected in the recruitment questionnaire included the cat owner’s location in New Zealand, when their cat was euthanized, why the euthanasia was performed and location of their veterinarian. Owner demographic information collected included age range, ethnic group, and gender. Purposive sampling [48,51,54] was then used to select owner participants who had had their cat euthanized within the last 3 months. Convenience sampling [48] was also used to select participants who were located in proximity to one another to facilitate the in-person interviews.

The number of owners interviewed was dictated by theme saturation. That is, we continued to interview owners until no new information was being mentioned. This resulted in 14 interviews.

### 2.3. Interview Structure

The first author undertook semi-structured single interviews with cat owners to explore how EoL decisions were made and how the process of euthanasia was managed. The role of the veterinarian was also explored. Interviews followed open-ended questions from an interview guide (Supplementary Materials). The questions were not made available to participants before their interview. The interview approach was conversational and relaxed, with follow-up questions and discussion. Interviews were digitally audio recorded, transcribed intelligent verbatim by a professional transcriptionist, and reviewed by the first author to ensure quality and accuracy. The names of owners, veterinarians, and veterinary clinics were replaced with numeric (e.g., ‘owner 1′) or other non-identifiable descriptors (e.g., ‘veterinary clinic’) for the purposes of data reporting.

### 2.4. Data Analysis

Transcripts were explored for themes using template analysis in NVivo qualitative analysis software [55,56,57]. Template analysis is a highly structured form of thematic analysis which emphasizes hierarchical coding and the development of a coding template [56]. This coding template is created using a subset of data, applied to further data, revised and refined. However, template analysis retains the flexibility to adapt to the needs of a study [55,56]. An example of this flexibility occurs when a template of preliminary codes is developed from initial interview transcripts, but these codes and their relationship to each other change as subsequent transcripts are analyzed. Open coding was used first; codes were created for interviewee comments that reflected their own words [58]. During an iterative reflective process, these codes were converted into themes that reflected the meaning behind interviewees’ words and relationships between themes. 

### 2.5. Terminology

Animal welfare status reflects overall well-being and is used to describe the mental state within an animal and the sum of their total experiences [59,60,61]. QoL and animal welfare status are largely thought of as synonymous terms [59,62,63]. We have used QoL throughout this article as it tends to be more easily understood by lay animal carers and reflects the vocabulary used by our participants [62].

## 3. Results

The final thematic template with high-level themes is presented in Table 1. There were two main categories of themes relating to considerations when owners decided to end the life of their cat: animal centered and human centered. Animal-centered themes were focused on the cat itself and how owners judged its QoL. Human-centered themes included considerations of all humans in the cat’s and owner’s life such as the veterinarian, family, and friends. The integrative theme of ‘prognosis’ was an aspect of many of the themes and reflected a need for the owner to predict what would happen to their cat. 

In the section that follows, direct quotes from owners are in italics. Square brackets are additional words inserted by the authors to clarify the meaning of a quotation. Punctuation has also been added to help the reader. A set of three periods indicates where filler words have been removed from the quotation.

### 3.1. Animal-Centered Themes

The four animal-centered themes (Table 1) identified from the data related to: cat behavior change, pain, signs of ageing, and the benefits of having other people see what owners often could not. Briefly, owners often mentioned their cat’s eating habits and other changes in their behavior. Food-related behaviors were clearly visible to owners, whereas judgements about pain were challenging for them. It was important to owners that their cat be pain free, but they found pain difficult to recognize. Owners also struggled to distinguish normal ageing from a decline in QoL that represented a need for euthanasia.

#### 3.1.1. My Cat’s Behavior Changed

Cat owners described a range of eating behaviors and focused on them throughout our discussions. Most owners found that their cat went off food towards the end of its life. When a cat went off its favorite food, a euthanasia decision soon followed. Owners were encouraging their cats to eat by the use of an appetite stimulant, hand feeding, giving them their favorite foods and treats, or by offering a range of options. A few owners resorted to forcing their cat to eat, particularly if they had not eaten for some time:

*“Which I didn’t mind doing for the greater good if he was gonna pick up and come round. But that went on for several weeks and he was getting worse and worse and fighting me…in the end it becomes torture.”*

In addition to changes in feeding behavior, most owners noticed that their cat had lost weight. This could have been despite the cat eating adequate or plentiful food. However, more often, the weight loss was related to lost appetite, despite the owner’s best efforts to encourage eating. 

Most owners also saw a reduction in overall activity in their cat. Cats spent more time in bed sleeping, or were just generally moving less:

*“...we would go to work and come home, and he would probably be in the same spot and he might get up for dinner?”*

Some cats were toileting in inappropriate locations such as in their own bed, areas in the home, or sometimes on the owner. Some cats stopped seeking their owner’s attention and did not want to be picked up, while others sought out their owners more:

*“And he wanted to be with you all the time. So, every time you sat down, you’d end up [with] a cat on your lap…And he’d stay there for hours if he could so [he] obviously didn’t feel very well.”*

Cats also communicated with owners differently; some became quiet, stopped purring, or held their tail down. Some cats vocalized more as they aged or became sick:

*“Towards the end he was really, really yowly. He…didn’t understand that he’d only been fed…ten minutes ago…he wasn’t there, and he was miserable.”*

Conversely, if their cat’s behavior had not changed, despite its age or serious disease, this was encouraging for most owners. Cats that were playing normally, hunting, purring, or still friendly towards their owners were perceived to be faring well. Once this changed, EoL decisions were not far behind. A behavior change, and particularly reduced appetite or inappropriate toileting behaviors, represented a poor prognosis for cats in the eyes of their owners.

#### 3.1.2. Pain Was Bad for My Cat 

Owners recognized that being pain free was an important aspect of quality of life. However, it became apparent that, while the presence of pain was important for EoL decisions, signs of pain were poorly recognized by owners. This created a tension between what owners’ thought was important, and represented a poor prognosis, and what they were seeing in their own cat:

*“…if I’d have thought he was in real pain I would really have done something a lot sooner”*

Many owners mentioned behaviors that were indicative of arthritis, such as stiffness around the back end, and linked these behaviors to pain experiences for their cat. However, it was not always clear whether they had made this link themselves or had been told their cat was arthritic by their veterinarian. One-third of cats were given analgesia, and for most cats this was initiated by their veterinarian, rather than by the owner themselves:

*“...this is the point at which we…really should think about putting him down and we took him to the vet...and they said ‘oh! no no it’s just it’s just a bit of…pain’ we gave him an injection of...pain killer and he perked up.”*

#### 3.1.3. Signs of Ageing in Cats Are Not Good

Owners did not think their cat’s age impacted on their decision to end its life; however, signs of ageing were perceived negatively by the owners we spoke with:

*“He was actually still really, really good. He slept a bit more and that was about it. But he didn’t really start acting old for a long, long time”*

Further, one of the younger cats in this study was negatively described as ‘ageing’ by comparing to an older cat they used to have:

*“...we just saw her grow into this really old cat very quickly over a matter of weeks…”*

Owners often linked ‘ageing’, whether real or imagined, to poor coat quality, body condition, or senility, for example:

*“...he was getting skinnier and skinnier and you know how old cats get.”*

Conversely, if an older cat was still playing normally, they were described to be young at heart:

“Well I was utterly outraged the first time I took [my cat] to the vet and they declared that he was geriatric. Cause he was still running around like a kitten and stuff. He was a very young old cat until…right up to the end”

Seven of the 13 cats in our study had been diagnosed with (or owners were under the impression they had) one or more diseases commonly found in older cats—for example, their kidneys or thyroid gland were poorly functioning. 

#### 3.1.4. Other People Could See What I Could Not

Owners found it hard to detect changes in their cat if they deteriorated slowly. This added an element of uncertainty about the cat’s prognosis. One owner emphasized the importance of another person’s fresh perspective:

*“Because when you live with something you don’t notice the changes as much, so it was [daughter name]’s comments more than anything I think.”*

Owners perceptions of chronicity were not always accurate and deteriorations in their cat were often hard for owners to see when it was a slow decline:

*“I was talking to my husband because I was just like ‘oh it’s so quick’. But he was like ‘well actually if you think back, it was a bit gradual’”*

Often it was this slow decline that made it hard to make an EoL decision or know when to draw the line on the cat’s life. Further, a fresh set of eyes, from someone who had not seen the cat recently, helped with evaluating how they are doing:

*“I don’t see it because I’m living with it every day. So, it was the gradual deterioration that when my friend [friend name] came and she saw the change…I just hadn’t noticed...It had kind of crept up on me…”*

### 3.2. Human-Centered Themes

Owners conveyed five over-arching human-centered themes (Table 1) relating to: veterinarians understanding owners’ relationships with their cats, normalizing death, the need for a good vet to manage EoL, veterinary validation that owners were doing the right thing, and a strong desire to predict the time course and outcome for their cat. 

#### 3.2.1. Understand My Relationship with My Cat

The majority of owners were very dedicated to their cat. They put their animal’s interests first and felt a deep connection with the cat to the extent that they described their cat as a member of their family or a friend. More than one-third of the cat owners I spoke to referred to their cats as children. One owner described herself as a stay-at-home parent. She worked from home to be with her “*children*”. It was not until 20 minutes into the interview that I realized the children she was referring to were her cats. Her positive view of her veterinarian was the result of him understanding the value she placed on her cats, her relationship with them, and her personal circumstances:

*“I felt that he genuinely cared. And I felt that he genuinely wanted the best thing...I wasn’t another file. I felt that it was he genuinely wanted the best interests of my child”*

This was in contrast to a previous veterinarian who had not understood this close parent–child relationship between owner and cat. 

There was obvious overlap between how owners perceived their cats, particularly as family members or children, and their responsibility to animals. For example, owners mentioned that they were willing *“to do what it costs”* for their cat, that the animal should come first, and that *“animals are forever”*. 

Generally, our participants were dedicated cat owners. It was challenging for them when other people in their lives did not understand this relationship. Comments from others reflected a different value placed on cats:

*“My brother would go ‘it’s just a bloody cat’…And I went off my nut at him once about that when he said ‘it’s just a cat what are you worried about just get another one’…It’s like you know what my cats mean to me they are my children and I don’t care that to you it’s just an animal”*

Comments like this one, about the owner’s relationship with their cat, made owners reflect on the support they received:

*“So, you’re not able to seek that support externally because other people don’t understand the relationship that you have with your animal”*

In other words, there was an expectation for support and acknowledgement of grief following the euthanasia by friends and family, despite them not feeling the same way about animals as companions or members of the family.

Three of the owners were diagnosed with cancer prior to or at the time of their cat’s euthanasia. This significantly changed the owner’s relationship with their cat and so influenced the cat’s life at the end. Owners recuperating from their own treatments at home with their cat reported becoming very attached to them and coming to rely on their company. Taking care of their cats often gave owners something to do or think about to distract from their own situation:

*“Cause I’m off work at the moment now with my own illness. So, I’d just spend the day looking after him and feeding him. Trying to hand feed him just constantly...So the two of us just spend the day recuperating. So, he’d come and snuggle on the chair with me.”*

These owners felt a deep connection with their cat as a result of this shared experience, with the effect of making the EoL decision much more difficult for them. One owner was undergoing chemotherapy treatment for terminal cancer herself at the time of her cat’s decline and this personal experience affected her choices. Delaying her cat’s euthanasia was viewed as a way of delaying more loss in a family who were already grieving for her:

*“...there’s too much death in the household…If it had been the previous year, I would have done it earlier rather than later...I just wasn’t strong enough. Neither of us were. It was just too hard”*

In contrast to these dedicated owners, some owners had a casual relationship with animals, or this cat in particular. They may not have been as close to this cat as others they had owned, or they chose to euthanize this cat earlier than they may otherwise have for their own perceived benefit.

#### 3.2.2. Death Is Normal

The owner’s attitude to, and experience with, death also influenced their decision to end their cat’s life. Death was normalized by owners and was described as natural by one owner:

*“...there’s nothing yucky about it. There’s no blood there’s no gore. It seems very natural. As natural as you can possible make it.”*

Other owners normalized death for their children by involving them in the cat’s death. Most owners believed death was a kindness for animals because they spoke about death and killing as *“humane”* or *“the kindest thing”*. For many owners, they rationalized the decision to end the life of their cat by not wanting to see them suffering:

*“The main deciding factor was that…I didn’t want him to suffer. And it wasn’t fair on him at all…I wasn’t going to keep him alive for us. Cause that’s so not fair. And I just looked at him and we thought you’re not happy”*

One owner felt the cat took the decision out of their hands, that there was no point in keeping the cat alive, and that the decision had to be made as soon as possible:

*“I was in denial for quite a bit. And then when it got kind of obvious...It wasn’t a hard decision. It wasn’t a decision at all really”*

Once experienced, this feeling of not having any other choice helped owners make the decision to end their cat’s life *“I think it’s maybe easier when it’s obvious that they’re not living as well as they have been”*. Owners did not want to prolong the life of their cats unnecessarily *“better a week too early than a day too late”* and felt that euthanasia was part of the responsibility of having an animal.

*“You know where you’re going with this and you know that it’s not recoverable. You know that it’s only gonna get worse. You just have to decide where on the slope [of QoL decline] you make the decision.”*

Owners compared the death of animals with the death of humans, and particularly loved ones who they had watched suffer while dying. Many owners thought that death could be dignified. One-third of owners were upset that they could make the decision to end the life of their cat but had to watch human loved ones suffer while dying:

*“It’s not a nice process but I wish that I could have done that to my mother…So I think it’s interesting how we can give animals an ending that I would wish that on the people that I love the most…I would want someone to put me out of my suffering”*

*“...you just don’t want to have to make that decision. But at the end of the day you’ve got to make it for them…That’s the thing we can do that for our animals, but we can’t do that for our people…”*

Despite normalizing death, two-thirds of owners had difficulty facing their cat’s death. They did not want to give up on their cat and wonder ‘what if’ they recovered. This was particularly problematic if the prognosis for their cat was unclear. As a result, these owners gave their cat a chance or kept them comfortable until they could make a decision. 

#### 3.2.3. I Need a Good Vet

Owners’ opinions of their veterinarian were important for how they related to them and how the veterinarian helped with EoL decision making. Most owners knew their veterinarian well because they considered them to be a ‘good vet’ and so had returned to them many times. Other things that made their veterinarian good in the owner’s eyes were whether they felt their veterinarian understood them or genuinely cared. Veterinary empathy resulted in a trusting relationship between owner and veterinarian:

*“Oh her opinion is probably the only one I would listen to on the welfare of my cats because I trust her to have their best interests at heart…My vet is not in it for the money, my vet’s in it for the animals…I have had dealings with other vets here that were not particularly good. And that was the catalyst that made me put more of my trust in this particular vet. And having known her and grown a relationship”*

Owners also highly valued their veterinarian’s relationship with their cat and described their veterinarian positively if they appeared to get along with their cat. Owners were also clear that it was their choice whether to euthanize their cat or not, but others did play a role in the decision-making process. Owners were not happy with their veterinarian if their values or opinion about euthanasia did not align with their own, or if they came across as inexperienced, by referring to their notes too much or otherwise appearing like they did not know what they were doing.

Seeing different veterinarians for problems with the same animal made the decision-making process harder for owners. Not only did they not have that close relationship with the veterinarian, but they also had to relate to them all differently and explain the cat’s condition over again to different people. Thus, continuity of care was important to owners and many of them insisted on seeing the same veterinarian each time or found a clinic where they saw only one veterinarian. However, some owners were not aware that they could request a certain veterinarian, and without being offered the choice, they took what they were given:

*“…they don’t often ask me when I ring up for an appointment ‘oh which vet do you want?’. It’s basically if you want an appointment at this time or this day you basically get who you get”*

According to owners, veterinarians could help them grieve by their actions or interactions. Sending cards or flowers after the loss of their cat was a small action that greatly impacted owners. Many owners wanted veterinarians to have a role in dealing with their grief. Training to deal with grieving clients, understanding their clients, better communications skills, and compassion fatigue training were variously mentioned by owners as ways that veterinarians could better deal with owners following pet loss:

*“I think it’s not just medical and professional management of the animal. There’s also an element of social worker in there because you’re dealing with a grieving owner who can possibly get upset or angry”*

Owners also expected veterinarians to be good at performing euthanasia. They felt the veterinarian should be familiar with the euthanasia procedure, have seen euthanasia performed before, be able to perform euthanasia smoothly, know what to do if things go wrong, and be supervised if still learning. 

#### 3.2.4. Tell Me I Am Doing the Right Thing

The veterinarian’s opinion about their cat’s prognosis was important to owners. The most important role the veterinarian had in the minds of the owners we spoke with was one of validation. One owner articulated this role perfectly when she told us she already knew what her veterinarian was going to say when asked, but that she was just reaching out for a second opinion on doing the right thing:

*“I think you go to your vet because, even though in your heart you know, you still want the professional to agree with you”*

Owners appreciated this validation, and it often helped with the grieving process to hear that the veterinarian, as the expert or professional, agreed with them:

*“…as soon as he saw him, he was just like ‘no you’ve done the exact right thing’. And he said it and that helped…that really really helped you know to kind of validate it”*

Even if they did not validate their decision, their veterinarian’s opinion was important to owners as they were the expert or professional in this situation:

*“…you’re with a professional to get their professional opinion. What you do with it is your decision to make…The reason why you’re there is because you want that expert opinion”*

Other roles taken by veterinarians were guiding owners towards euthanizing their cat by using subtle comments to encourage euthanasia (e.g., asking owner to think about what is best for cat or talking about doing the right thing), preparing owners for the worst by discussing the cat’s compromised QoL, or explicitly telling the owner to euthanize their cat. 

#### 3.2.5. Tell Me What Will Happen

Because we were talking to owners about the whole process leading up to the euthanasia of their cat, they mentioned times when they did not necessarily see death as the outcome. Owners struggled if the outcome for their cat was unclear or unpredictable. They were unable to process not knowing how long their cat had to live, or what they might go through towards the end of their life. As a result of this underlying difficulty, owners asked their veterinarian how much longer their cat had or consulted the internet for advice on their cat’s condition:

*“I’d gotten, as you do, Dr Google. I’d gotten online and started googling you know ‘latest stages of renal failure’…I found a really good article…”*

One owner was not aware her cat’s condition was terminal until near the very end of its life and at least half of the owners we spoke with were hopeful their cat would improve:

*“So the last kind of 24 to 48 h were like last ditch efforts you know…And it was like if none of this works that’s it…So I left him overnight to see if he’d pick up and he just didn’t”*

A number of owners appreciated honesty from their veterinarian and asked the question: *“if it was your cat what would you do?”*. This owner respected her veterinarian for directly answering her question:

*“And she will bluntly tell us…A spade’s a spade with her ‘…I can’t guarantee that the reconstruction surgery is going to fix him. To be perfectly honest I would put him to sleep because he’s not really going to have a very good life, even if we do manage to keep him alive’…”*

Knowing what would happen to their cat was important for owners, and veterinarians who were honest with them were perceived as good veterinarians. Without this knowledge, owners were lost and many of them resorted to asking ‘Dr Google’.

## 4. Discussion

Ending the life of an animal is difficult for many owners. When that animal is a beloved family member, the owner’s decision is not taken lightly, and there are additional considerations for veterinarians to make. In this study, owner considerations when making the decision to end the life of their cat were grouped under two main themes: animal centered and human centered. Each theme will be discussed alongside the veterinarian’s role, real or potential, in helping owners deal with their concerns.

### 4.1. Animal-Centered Themes

When they explicitly focused on their animal, cat owners described their cat’s behavior and physical appearance. Weight loss was perceived as a bad sign by owners and it accelerated their decision. When their cat went off their favorite food, an EoL decision closely followed. 

Changes in weight and appetite are important considerations in QoL assessment protocols, and particularly in those designed for EoL decision making [64,65]. There are a number of QoL assessment tools available for owners and veterinarians to use including those designed for use with dogs and cats [66,67] and those specific to cats [68,69]. 

Weight loss is a robust indicator of an animal’s nutritional status [70]. Therefore, it follows that it played a significant role in decision making for our owners. It is also measurable and quantifiable—both of which help when making difficult decisions such as ending the life of an animal. Appetite can also be easily observed by owners and is quantifiable in cats fed in a controlled manner (e.g., indoor only). However, weight loss does not always accurately reflect welfare status [62], and it can be challenging for owners to quantify without ready access to a scale. Appetite is also difficult to measure when cats have outdoor access and are free to eat indiscriminately, as is the case for most New Zealand cats [2].

Repeated measures of a cat’s weight over time and discussion of appetite can help owners and veterinarians assess QoL [71,72]. Multi-buy deals for weigh-ins and visits with a nurse technician to discuss feline QoL, with follow-up appointments with a veterinarian, could help. These visits could be offered from the start of an animal’s life or begin when the animal is diagnosed with a terminal illness or falls into the ‘geriatric’ category. This assistance could have the added benefit of veterinary professionals being perceived as ‘good vets’ and help ‘tell [owners] what will happen’. Problems may arise with cats that have a negative association with the veterinary clinic, or whose owner perceives that they do. These cats could be counter-conditioned to veterinary visits or offered a home visit [73]. The recent upsurge in mobile veterinary practices could help improve feline veterinary care in this regard [34,36,74,75,76].

Pain or signs of ageing were perceived to be bad for cats, but owners grappled with how to evaluate pain and how to distinguish normal ageing from poor QoL. Pain also features in QoL assessment protocols, and particularly in those designed for EoL decisions [64,65]. For example, one such protocol assigns low “Ouch or Pain” 10 of the available 80 QoL assessment points towards “a healthy and happy pet” [64].

Pain is often framed as being treatable and only intractable pain warrants euthanasia [64,65,77]. However, at least two problems arise from this stance: (1) it ignores positive life experiences and the potential for analgesia to impact on these, that is, via sedation or other side-effects impacting the animal’s QoL; and (2) pain is often poorly recognized. In common with the owners in our study, other authors have reported that, despite numerous pain-specific assessment protocols available [78,79,80], pain in cats is often difficult to assess [81,82,83]. Above all, pain and its management at the end of animal’s lives should be critically considered in the context of the animal’s overall QoL, that is, to consider euthanasia as an option earlier.

Animal age rarely features in EoL decision-making protocols. Advanced age is often cited as a reason not to euthanize an animal, i.e., that their clinical signs are a result of the normal ageing process, rather than declining QoL [65]. Nearly half of the cats in our study had been diagnosed with a disease commonly found in older cats, e.g., hyperthyroidism or kidney dysfunction [8,84,85]. This would have made it difficult for owners to distinguish between an age-related decline in QoL, and a decline resulting from one of these diseases. 

Our owners considered their veterinarian to be the professional or expert in EoL decision making and euthanasia. To fully realize this role, veterinarians need to be skilled at pain recognition and effectively communicate the results of pain evaluations to their clients. In addition to pain, we need to be clear that there are other unpleasant experiences associated with ageing or chronic conditions in animals—for example, nausea resulting from kidney dysfunction. These experiences are no less important to the animal’s QoL. Veterinary–client communication could also take the form of resources to help owners assess their animal’s progress—for example, QoL assessment protocols. An open discussion about QoL, its assessment, and the current status of their animal is also an option [71,72]. 

Owners in our study may not have always understood that their cat was on a trajectory towards death when they first presented their cat to their veterinarian. Therefore, veterinarians need to be aware that owners presenting an older animal, or one with a chronic disease, do not always realize they might die soon [40]. This gives additional credence to veterinarians having early QoL conversations with their clients. 

### 4.2. Human-Centered Themes

The cat’s QoL was not the only consideration for owners in our study. Veterinarians needed to understand that a euthanasia decision meant permanently breaking a deep connection an owner had with their cat. This deep connection, gradual deterioration, and lack of distance to recognize change often blinded owners to their cat’s condition at the time and they needed someone else to help them see how their cat was really doing. Many of our owners saw their cats as family members, and this is echoed in other studies, where it may be linked to strong attachment to animals [14,74,81]. 

Most of our owners could normalize death in some way and euthanasia was often perceived to be a dignified ending. However, owners still wanted their veterinarian to know how difficult death was for them. This conflict between normalizing death, and yet struggling to come to terms with it, could result in owners prolonging the decision to euthanize their cat. A number of our owners admitted this being the case for them. 

Keeping an animal comfortable until a decision is made is one of the cornerstones of palliation during veterinary hospice care [85,86]. This can be problematic when the cat has little to no chance of recovery and their QoL is severely compromised [14,81,85]. In these cases, the owner may be prolonging the life of their cat for their own benefit, that is, as a result of anticipatory grief [14,29]. This creates an ethically challenging situation for the veterinarian whose responsibility lies with upholding the QoL of the animals under their care [11,12] and yet has some obligation to their paying animal-owning client. Added to this, animals are considered legal property of their owners in most jurisdictions [22]. However, some allowances are made for veterinarians to intervene when necessary. For example, Section 138 of the New Zealand Animal Welfare Act 1999 states that veterinarians “must, without delay, destroy that [severely injured or sick animal] animal or cause it to be destroyed” if the animal’s owner does not agree to its destruction and does not seek a second opinion in a reasonable time [22]. This emphasizes the existing belief that veterinarians need training in ethical reasoning and communication skills during their degree or as continued professional development after graduation [87,88,89].

Prognosis was an integrative theme that appeared throughout our discussions with owners. Owners wanted to predict the time course and outcome for their cat and struggled with the EoL decision when the ending was unclear. Some of our owners resorted to asking the internet for help instead of their veterinarian. This may have been because they felt their veterinarian was not involved in the decision or because they wanted to gather more information to make an informed decision. Whatever the case, it behooves the veterinarian to ensure the information that clients are using to make a decision and that will impact the welfare of an animal under their care, is accurate, reliable, and timely. As other authors have suggested, this information could be provided by the veterinarian in the form of literature to read or by giving owners validated and reliable QoL assessment protocols to use for their cat [62,72].

Most owners considered their veterinarian to be a ‘good vet’ and so had returned to them multiple times with their animal. This was valuable during the EoL decision-making process because they already had a trusting relationship with their veterinarian. Conversely, owners did not like it when they saw different or inexperienced veterinarians for their cat’s condition. Continuity of care is important for clients, particularly when they are making important decisions about their animal’s well-being [11,44,90]. The ‘good vet’ would know them and their animal, tell them they were doing the right thing for their animal, and validate their decision. 

Similar to findings by Christiansen et al. [40], owners in our study had different preferences for their veterinarian’s role in EoL decision making. These ranged from the desire for validation of their own decision, considering their veterinarian an expert and wanting to know what would happen with their cat, through to asking the veterinarian what they would do. Some owners in the aforementioned study wanted information and professional assessments from their veterinarian, others wanted support, while still others wanted their veterinarian to legitimise their decision, and a subset of the owners wanted their veterinarian to guide them into a decision [40]. It is clear, from these results, that there are a range of preferences for owner–veterinarian interactions during EoL decision making and that ongoing and trusting relationships allow veterinarians to provide appropriate support.

Veterinarians can have various roles when they interact with their clients. Examples of these roles include the paternalistic veterinarian who tells the owner what to do [91], the information provider who leaves the decision to the owner and respects their autonomy, or the veterinarian who influences their client when their choices are not ‘reasonable’ [40,92]. The paternalistic model of health care contrasts with client autonomy and informed choice and has since gone out of favor in both human and veterinary medicine [90,91,93]. Veterinarians can also share in the decision-making process with their client [40,93], or can exert their Aesculapian authority [93,94]. They can act as garage mechanics or akin to pediatricians [93,94]. The Strong Patient Advocate (SPA) concept recognizes that veterinarians are obligated to their patient, i.e., the animal under their care, and aligns with Codes of Professional Conduct for Veterinarians [11,12,95]. 

‘Client autonomy’ is often touted in the veterinary literature as being Best Practice for owner–veterinarian interactions during EoL decision making [96,97]. The term regularly appears in North American-centric publications and is often defined as the veterinarian giving the owner the information to make an informed choice for their animal [97]. However, an alternative option for owner–veterinarian interactions places the onus on the client to decide how much of a role their veterinarian plays in the decision-making process [40,92]. The cat owners in our study were clear that it was their choice to euthanize their cat, but also highly valued their trusted veterinarian’s opinion and acknowledged that other people did play a role deciding. Yeates and Main [92] argue veterinarians can exert influence over their client’s decision making in circumstances when the client has asked them to. In this case, veterinarians may lead the decision-making process, while still respecting client autonomy, that is, because the client has delegated the veterinarian this role [92,98].

Overall, it was apparent that a ‘good vet’ needs to understand the owner’s personal situation, their preference for veterinary assistance with decision making, and validate their decision at the end [29,36,40]. The veterinarian could take on a number of different roles, dictated by the client, to achieve this, and preferably, the owner would deal with the same veterinarian during the EoL decision-making process. This ongoing, trust-based relationship would facilitate the veterinarian fulfilling the role desired by a particular client. 

### 4.3. Study Limitations

This small-scale study was conducted using social science methodology with qualitative data. As such, generalizations to the wider cat-owning community of New Zealand cannot be made [29,45]. However, this study provides valuable information regarding the ways in which EoL decisions are currently being made by owners of older and chronically ill cats in New Zealand and the role of their veterinarian in the process. A major limitation of the findings is that our owner participants were those who presented their cat for euthanasia by a veterinarian. This excludes those who left their cat to die ‘naturally’ or killed their cat themselves. In common with other studies in this area, our owners were also largely dedicated owners with strong attachments to their cats [28,29]. This is probably because these are the owners who felt strongly enough to share their experiences with researchers. However, these owners are probably the ones most in need of veterinary input as they struggled to make the decision to end the life of their family member.

Most of the euthanasia events occurred within three months of the interview, and all were no more than six months before. However, as others have found, there may still have been recall bias in our participants. For some, this bias will have been compounded by them attempting to remember what may have been a traumatic event [29].

Overall, our results should be applied and understood in the context of the methods we used to collect the data. Our participants were New Zealand owners of older cats and those with chronic disease who were willing to share their experiences of the decision-making process with a researcher and were also happy for their veterinarian to be approached for interview. This suggests that they had a reasonable relationship with their veterinarian.

This study is one of few that addresses the challenges inherent in owner decision making for their cats, and the only study, to our knowledge, that does so using a qualitative methodology in the form of in-person client interviews.

## 5. Conclusions

Most owners in our study struggled to make the decision to end the life of their cat. Many of the owners realized that their cat’s welfare was poor but were not sure where on ‘the slope’ [of QoL decline] they should make the decision to end its life. Owners were often torn between wanting to give their cat a good chance to recover and not letting them suffer if they did not have long to live. They emphasized a need to predict what the time course and outcome would be for their cat. Their veterinarian was key to both validating and informing owner EoL decisions.

EoL decision making is complex and veterinarians often find their role poorly defined [99]. However, our owners appreciated the validation and expertise that their veterinarian offered them as well as their understanding of their relationship with their cat. Veterinary practices could help owners deal with this difficult situation by offering their clients repeat visits with the same veterinarian who understands their needs, i.e., continuity of care. Future research aimed at exploring the veterinarian’s perspective during EoL decision making for the same animal would be a valuable addition to the field.

## Figures and Tables

**Table 1:** Final template for owner interview themes.

1. Animal-Centered Themes			
1.1 My Cat’s Behavior Changed			
	1.1.1 Eating behavior changed		
		1.1.1.1 Cat was eating and drinking	
		1.1.1.2 Cat went off food	
		1.1.1.3 Cat was encouraged or forced to eat	
		1.1.1.4 Cat was always hungry	
		1.1.1.5 Cat lost weight	
	1.1.2 Cat’s interactions changed		
		1.1.2.1 Cat’s interactions with its surroundings changed	
		1.1.2.2 Cat’s interactions with other animals changed	
		1.1.2.3 Cat’s interactions with humans changed	
**1.2 Pain Was Bad for My Cat**			
	1.2.1 Pain is important		
	1.2.2 Cat had signs of arthritis		
	1.2.3 Cat was given analgesia		
	1.2.4 The non-painful cat		
**1.3 Signs of Ageing in Cats Are Not Good**			
	1.3.1 Cat had poor coat quality or body condition		
	1.3.2 Cat had signs of cognitive decline		
	1.3.3 Young cat ‘aged’ quickly when sick		
	1.3.4 Cat had disease of old cats		
	1.3.5 Young at heart		
**1.4 Other People Could See What I Could Not**			
	1.4.1 Owner discussed cat with other people		
	1.4.2 Other people noticed cat’s condition		
**2. Human-Centered Themes**			
**2.1 Understand My Relationship with My Cat**			
	2.1.1 Owner was dedicated to cat		
		2.1.1.1 Owner put animal’s interests first	
		2.1.1.2 Owner felt deep connection with animals	
	2.1.2 Owner had casual relationship with cat		
		2.1.2.1 Owner was not as close to this cat as others	
		2.1.2.2 Owner euthanized cat earlier for own benefit	
		2.1.2.3 Cat needed more care than owner could offer	
	2.1.3 Owner felt their veterinarian understood their relationship with cat		
**2.2 Death Is Normal**			
	2.2.1 Death is normal		
		2.2.1.1 Death is natural	
		2.2.1.2 Owner was prepared for cat’s death	
		2.2.1.3 Owner had experience with animal death	
	2.2.2 Death is a kindness		
	2.2.3 Death is difficult and final		
		2.2.3.1 Owner did not want to give up on cat	
		2.2.3.2 Owner delayed euthanasia for own benefit	
		2.2.3.3 Owner struggled with euthanasia event	
		2.2.3.4 Cat had ‘The Big C’ [cancer]	
		2.2.3.5 Euthanasia is owner’s decision to make	
		2.2.3.6 Others had different thoughts about animal death	
	2.2.4 Death can be dignified		
		2.2.4.1 Owner compared animal and human death	
		2.2.4.2 Owner brings up family member loss	
		2.2.4.3 Cat’s euthanasia was ‘good’	
**2.3 I Need a Good Vet**			
	2.3.1 Owner thought their veterinarian was a good vet		
		2.3.1.1 Owner knew veterinarian well	
		2.3.1.2 Vet genuinely cared	
		2.3.1.3 Vet had good relationship with cat	
	2.3.2 Owner was not happy with veterinarian		
		2.3.2.1 Vet was inexperienced	
		2.3.2.2 Vet’s values did not align with owner’s	
		2.3.2.3 Vet’s opinion about euthanasia differed to owner’s	
	2.3.3 Different veterinarians made it harder for owner		
	2.3.4 Veterinarians can help owners grieve		
		2.3.4.1 Vets need training in psychology	
		2.3.4.2 Vet’s actions helped owner grieve	
	2.3.5 Owners expect veterinarians to be good at euthanasia		
**2.4 Tell Me I Am Doing the Right Thing**			
	2.4.1 Veterinarian validated owner’s decision		
		2.4.1.1 Vet agreed with owner’s euthanasia decision	
		2.4.1.2 Vet is expert or professional	
	2.4.2 Veterinarian guided owner towards euthanizing cat		
		2.4.2.1 Decision making was shared with vet	
		2.4.2.2 Vet used subtle comments to encourage euthanasia	
		2.4.2.3 Vet prepared owner for worst	
		2.4.2.4 Vet told owner to euthanize cat	
	2.4.3 Veterinarian gave owner options		
		2.4.3.1 Vet gave owner treatment options	
	2.4.4 Veterinarian dissuaded owner		
	2.4.5 Veterinarian was confused or had no role in decision making		
		2.4.5.1 Owner made euthanasia decision before seeing vet	
		2.4.5.2 Vet did not tell owner to euthanize	
		2.4.5.3 Vet was unsure of their role	
**2.5 Tell Me What Will Happen**			
	2.5.1 Owners were tracking cat’s progress		
		2.5.1.1 Owner was recording information about cat	
		2.5.1.2 Owner was hopeful cat would improve	
		2.5.1.3 Cat deteriorated	
	2.5.2 What would you do?		
		2.5.2.1 Owners wanted their vet to be honest	
		2.5.2.2 Unclear expectations were difficult for owner	
		2.5.2.3 Vet did not tell owner what to expect with cat	
**3. Prognosis** (Integrative Theme)			

## Supplementary Materials

The following are available online at https://www.mdpi.com/article/10.3390/ani11041114/s1, Table S1: Interview guide.
