# Supplementary material for: “What Would You Do?”: How Cat Owners Make End-of-Life Decisions and Implications for Veterinary-Client Interactions
Source: Animals (Basel). 2021 Apr 13;11(4):1114. doi: 10.3390/ani11041114 (PMC8068809; doi:10.3390/ani11041114)
Supplement: Supplementary file 1 [file animals-11-01114-s001.pdf]

Retrospective Euthanasia Interviews

OWNER INTERVIEW

**TOPIC (estimated time in minutes)**<br>*Keywords*	**QUESTIONS [AND PROMPTS]**
**Introduce Interviewer & Research (10)**<br>*About interviewer and project*	Kat Littlewood, PhD Candidate at Massey University. Interview is part of PhD research.<br>We would like to find out how owners and their veterinarians are currently making EoL decisions.
Outline of interview procedure	I will first start off with some background questions.<br>Then I would like to discuss cats in general, before moving onto talking about your own experiences with your cat.<br>I would be happy to explain more after the interview.<br>I will ask number of questions - please answer what comes to mind. No right or wrong answers. Want to hear what you think.<br>I may take notes – so I don't interrupt you if something you say I would like to hear more about later in the interview.<br>If you think of something you think is important, but I don't ask about it, you are welcome to bring it up yourself.
Opportunity to withdraw, break	If I ask something you don't want to talk about, you can say you don't want to answer.<br>You can stop interview at any time.<br>If you need to take a break during the interview please say so.
Written transcript, summary report	You will have an opportunity to view the written transcript and make any corrections if you wish.<br>This may take up to one hour of your time, and we would expect the revised transcript to be returned to us within two weeks of you receiving it.<br>If we do not hear from you within this two week period we will assume that you have given permission to include the interview in the analysis.<br>If, after reviewing the transcript, you are happy with it as it is you do not need to return it.<br>You will also receive a summary of the project findings when it is concluded.
Consent	Information Sheet & questions.<br>**Consent Form signed**.<br>I would like to record interview – it will be transcribed afterwards, but your responses kept anonymous. OK to record interview? Can we begin?<br>**Start recorders.**

Probing questions: 'Can you tell me a bit more about that?' 'What was that like?' 'Can I check, you said x happened; how did you feel about that?' 'Why do you say that?'
Encouraging interesting material: 'That's really interesting', 'Then what happened?' **LEARN TO WAIT IN THE FACE OF SILENCE**

**TOPIC (estimated time in minutes)**<br>*Keywords*	**QUESTIONS [AND PROMPTS]**
**A: Warm up questions (2)**<br>*Experience with animals and children/family*	**What is your previous experience with animals?**<br>• Farm/livestock experience?<br>• Have you always had a cat? How many?<br>• Other pets/animals they have access to or experience with?<br>**Who is in your household?**<br>• Children? other animals?
**B: Experiences with euthanasia and death. Expectations of longevity (2)**	**How long do you expect cats in general to live for?**<br>**What do you consider an 'old cat' to be?**<br>• How long did you expect your own cat to live for? [before/after diagnosis – if applies]<br>**Was this your first experience with putting a pet down?**<br>• Have you ever had to put a pet down before this?
**C: About their cat (5)**	**Tell me a few things about your cat [name of cat] – cat's background**<br>• What was your cat's name?<br>• How old was s/he? [cats gender]<br>• When did you get her/him? [duration of ownership]<br>• Was s/he desexed/neutered?<br>**We are now going to focus on your <u>experiences</u> with your cat. Tell me about what was going on with your cat.**<br>• How did you find out s/he was ill? [or not doing so well]<br>• Do you remember when you first considered the possibility of putting your cat to sleep?
**D: <u>Why</u> did owner decide to euthanase cat? (timing)** Overall factors involved in decision e.g. decline QoL, etc. Was there a major factor?<br><br>How did animal's dx impact on owner's life?	**<u>Why</u> did you decide to put down your cat?**<br>• What **factors** involved in decision? – what was **most important factor**? Why?<br>• How **long** did it take for your pet to go downhill? [Did you have to make a **quick EoL decision**? [why/why not]]<br>• How **old** was s/he when you were given the diagnosis? Did the age of your cat factor into your decision?<br>• How much were you expecting your cat's treatment to **cost**? How much did the **cost** influence your decision?<br>• What was a **typical day** like for you and your cat after s/he became ill or started to decline? How was it different?<br>• Was there anything at home that you started to do differently or changed? How did you handle this?<br>• Were there any **sacrifices made** by your or your family for your cat?<br>SECTION G NOW
**E: What was vet's role in the EoL decision? (6)** <u>How</u> was EoL decision made?	**Who did you talk to about your decision? Whose advice** did you seek?<br>• Why did you ask this person?<br>• Do you remember any **reactions from other people** to your cat's illness or age?<br>**How important was your veterinarian's advice in your decision-making?**<br>• What **options** were you given by your veterinarian? Did they change how you decided?

Probing questions: 'Can you tell me a bit more about that?' 'What was that like?' 'Can I check, you said x happened; how did you feel about that?' 'Why do you say that?'
Encouraging interesting material: 'That's really interesting', 'Then what happened?' **LEARN TO WAIT IN THE FACE OF SILENCE**

**TOPIC (estimated time in minutes)**<br>*Keywords*	**QUESTIONS [AND PROMPTS]**
Who did they consult with to make this decision e.g. friends, family, internet groups, veterinarian	• What **role** did they have in your decision?<br>• How **long** have you known your veterinarian/practice?<br>• How much did your **relationship** with the veterinarian influence your decision?<br>• Do you feel that your **vet agreed** with your decision?
**F: The euthanasia event (3.5)**	**Can you tell me about the day that [cats name] died?**<br>• Tell me about the euthanasia event<br>• How did you find the euthanasia process? Any positive experiences?<br>• What was the decision-making experience like? What (if anything) could have helped to make that decision easier?
**G: How did the owner assess QoL/welfare before and leading up to this decision? (4.5)** E.g. not eating, not drinking, not as active. Are they making these decisions based on QoL assessments?	**How did you decide on/evaluate how your cat was doing? (Its QoL/welfare)**<br>• What does **good QoL** for your cat mean to you? How did you assess your cats QoL?<br>• What was the most important factor in your cat's QoL? [What did your cat enjoy? What was most important to him? Did this change? Did it affect decision?]<br>• How would you rate your **cats QoL in the last 2 weeks before** you put him/her to sleep? Why do you say that?<br>• How would you rate your **cats QoL in the last year before** you put him/her to sleep?<br>• What were your expectations regarding your cat's **diagnosis [prognosis] or his/her age and their life expectancy**?<br>**How did you discuss your cats QoL with your vet?**<br>• **When** did you start having these QoL discussions?<br>• Did your vet give you any like things that might happen or any idea of **things to look out for**?
**H: How attached is the owner to their pet? (1)** Will attach affect answers?	**How close to your cat were you?** What makes you say this?<br>• How did other people (friends/family) react to the loss of your cat? Did they understand your loss?
**I: Expectations of veterinarian (1.5)**	**What were your expectations of your veterinarian in terms of managing animal euthanasia?**<br>• How much knowledge and experience would you expect them to have to perform euthanasia?<br>• How important is it for vets to be taught EoL management (in vet schools)?
**J: Ending on a positive note (1.5)**	**What would be a 'good' pet euthanasia for you?** What does it mean for an animal to have been put down 'well'?
**K: Wrap Up**<br><br>*Possible additions, possible follow-up*<br><br><br>*Possible questions about the project*<br>*Difficult subject*<br>*Thanks*	It **seems like we're coming to the end** of our discussion, but I just want **to check my notes**… ('Could you say a bit more about…')<br><br>It seems we have covered main things I had in mind, but want to be sure you've told me everything you think I should know. Is there **anything you would like to add**? If you **remember something later**, please don't hesitate to contact me.<br><br>Do you have any questions about the PhD project or the further processing of information you have provided?<br>I understand this may have been difficult for you to talk about. **Are you going to be OK?**<br>You have been so helpful; I really appreciate the time you have taken to talk with me. Thank you very much.

Probing questions: 'Can you tell me a bit more about that?' 'What was that like?' 'Can I check, you said x happened; how did you feel about that?' 'Why do you say that?'
Encouraging interesting material: 'That's really interesting', 'Then what happened?' **LEARN TO WAIT IN THE FACE OF SILENCE**
